# Comparative analysis of maca (*Lepidium meyenii*) proteome profiles reveals insights into response mechanisms of herbal plants to high-temperature stress

**DOI:** 10.1186/s12870-020-02645-4

**Published:** 2020-09-16

**Authors:** Zhan Qi Wang, Qi Ming Zhao, Xueting Zhong, Li Xiao, Li Xuan Ma, Chou Fei Wu, Zhongshan Zhang, Li Qin Zhang, Yang Tian, Wei Fan

**Affiliations:** 1 Key Laboratory of Vector Biology and Pathogen Control of Zhejiang Province, College of Life Sciences, Huzhou University, Huzhou, 313000 China; 2 State Key Laboratory of Conservation and Utilization of Bio-resources in Yunnan, The Key Laboratory of Medicinal Plant Biology of Yunnan Province, National & Local Joint Engineering Research Center on Germplasm Innovation & Utilization of Chinese Medicinal Materials in Southwest China, Yunnan Agricultural University, Kunming, 650201 China; 3 College of Food Science and Technology, Yunnan Agricultural University, Kunming, 650201 China; 4 Huzhou central hospital, Huzhou University, Huzhou, 313000 China

**Keywords:** High-temperature stress, Maca, Molecular mechanism, Stress response, Tandem mass tag

## Abstract

**Background:**

High-temperature stress (HTS) is one of the main environmental stresses that limit plant growth and crop production in agricultural systems. Maca (*Lepidium meyenii*) is an important high-altitude herbaceous plant adapted to a wide range of environmental stimuli such as cold, strong wind and UV-B exposure. However, it is an extremely HTS-sensitive plant species. Thus far, there is limited information about gene/protein regulation and signaling pathways related to the heat stress responses in maca. In this study, proteome profiles of maca seedlings exposed to HTS for 12 h were investigated using a tandem mass tag (TMT)-based proteomic approach.

**Results:**

In total, 6966 proteins were identified, of which 300 showed significant alterations in expression following HTS. Bioinformatics analyses indicated that protein processing in endoplasmic reticulum was the most significantly up-regulated metabolic pathway following HTS. Quantitative RT-PCR (qRT-PCR) analysis showed that the expression levels of 19 genes encoding proteins mapped to this pathway were significantly up-regulated under HTS. These results show that protein processing in the endoplasmic reticulum may play a crucial role in the responses of maca to HTS.

**Conclusions:**

Our proteomic data can be a good resource for functional proteomics of maca and our results may provide useful insights into the molecular response mechanisms underlying herbal plants to HTS.

## Background

Maca (*Lepidium meyenii* Walp) is an herbal plant of the *Brassicaceae* family, natively cultivated in the central highlands of the Peruvian Andes [1]. Due to its potential health benefits and valuable medicinal properties, maca has generated great interest in pharmacological and nutritional research and been introduced to many places around the world, which make it an attractive plant for the nutraceutical industry in recent years [2]. Because it is cultivated at altitudes of up to 3500 to 4500 m, maca possesses robust tolerance to extreme environmental stresses such as cold, strong wind and UV-B exposure [1, 3]. Nevertheless, it is extremely sensitive to heat stress [1, 4]. Thus far, there is limited information about gene/protein regulation and signaling pathways related to the heat stress response (HSR) in maca.

Under climate change, high temperatures are believed to be a serious threat to crop yields due to their negative effects on plant growth and development [5]. In recent years, extreme temperatures have occurred more frequently and with more intensity due to global warming [6, 7]. High-temperature stress (HTS), which is also known as heat stress, is a complex function of temperature intensity, duration and rate of increase [8]. Under HTS, high temperatures frequently cause not only direct damage that includes protein denaturation, aggregation and increase in membrane lipid fluidity, but also indirect damage that includes inactivation of enzymes in chloroplasts and mitochondria, disruption of protein homeostasis, and loss of membrane integrity [9]. These damages further result in a decline in photosynthetic rate, disruption of water balance and protein homeostasis, decrease in ion flux, production of reactive oxygen species (ROS), and destruction of hormone levels and cell structure [8, 10]. These effects eventually result in growth inhibition and developmental retardation in plants [11, 12].

It has been shown that HSR-mediated tolerance mechanisms are key strategies to counter the effects of HTS on plants [5]. HSRs can raise the levels of numerous proteins that are produced from a specific set of HTS-responsive genes [13]. Therefore, the identification of proteins involved in HSRs is crucial to understand the molecular mechanisms of plant response strategies to HTS. To date, although several studies have investigated plant responses to HTS in tomato, grape, rice, and wheat using transcriptomic or proteomic approaches [8, 14–18], the molecular-level mechanisms underlying plant responses to HTS are still not fully understood, at least in herbal plants.

Tandem mass tag (TMT)-based proteomic analysis is a robust approach that extensively explores protein expression profiles and provides integrated information about individual proteins [19]. This advanced technology can be employed to determine the relative abundance of proteins between the control and treatment groups [20]. Over the past decade, the TMT-based proteomic approach has been broadly employed to explore differentially expressed proteins (DEPs) in plant development and stress responses [21–23]. However, this technique has not yet been applied to investigate the molecular mechanisms of the responses of herbal plants to HTS.

In this study, a TMT-based comparative proteome analysis of maca was carried out to explore the molecular mechanisms responsible for high-temperature responses. Based on these measurements and bioinformatics analysis, we found that the ‘protein processing in endoplasmic reticulum’ pathway was the most significantly up-regulated metabolic process following HTS. The transcription of genes encoding 19 proteins involved in this pathway was further examined by qRT-PCR and each mRNA was detected to be markedly up-regulated in maca seedlings exposed to HTS. These findings advance our understanding of crucial aspects of the molecular mechanisms underlying the responses to HTS in higher plants.

## Results and discussion

### Effects of HTS on morphology and physiology of maca seedlings

**Fig. 1:**
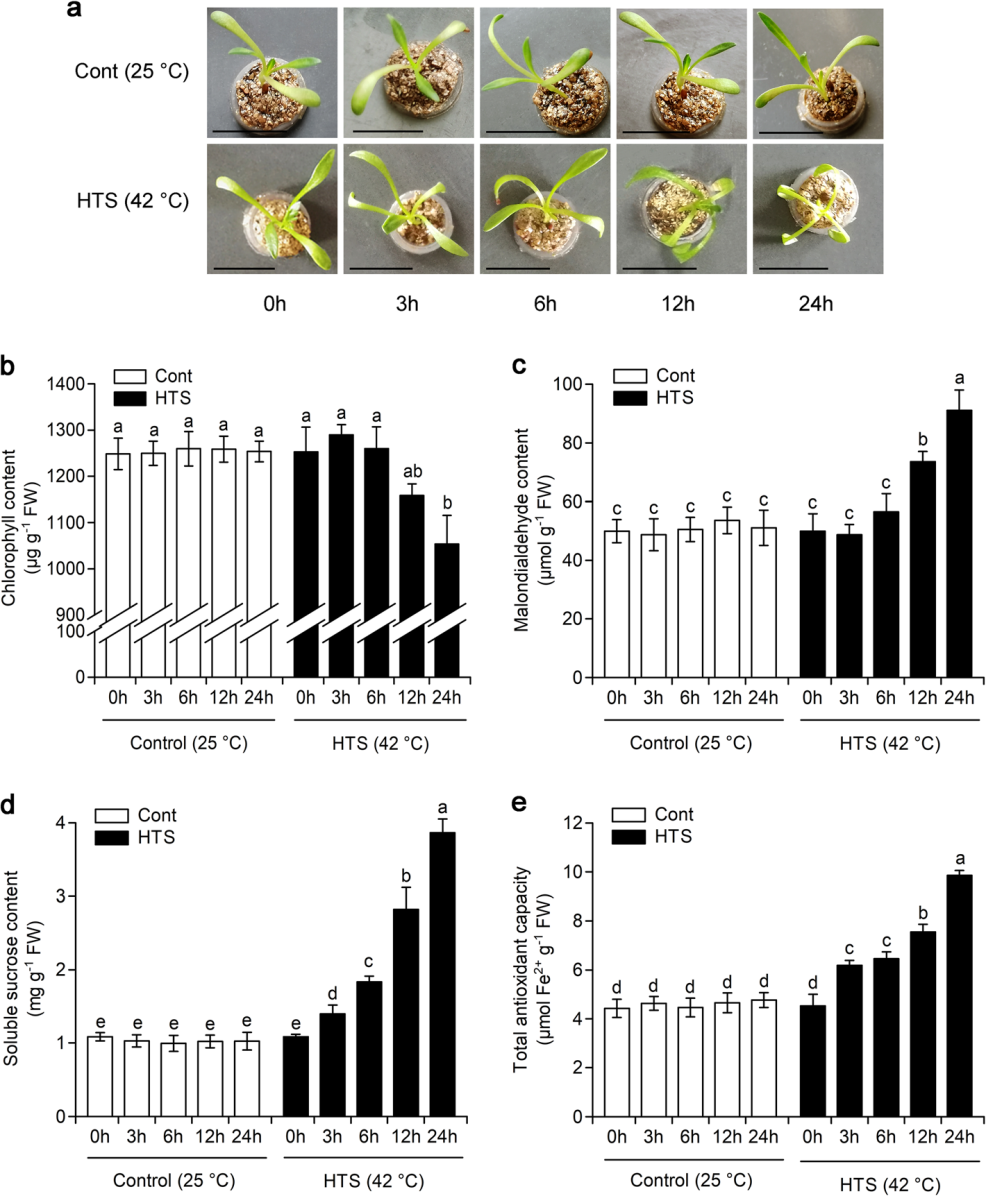
Effects of high-temperature stress (HTS) on morphology and physiology of two-week-old maca seedlings. **a** Phenotype of two-week-old maca seedlings under 42 °C for 0, 3, 6,12 or 24 h. Two-week-old maca seedlings grown at 25 °C for designated time points were used as controls. Bars, 1 cm. **b–e** Impact of HTS on (**b**) chlorophyll content, **c** malondialdehyde content, **d** soluble sucrose content, and (**e**) total antioxidant capacity in the leaves of maca seedlings. Two-week-old maca seedlings grown at 25 °C for designated time points were used as controls. Results are presented as means ± SD of three biological replicates and three technical replicates (*n* = 9). Different letters represent significant Student’s *t*-test differences at *P* < 0.05

Previous reports have shown that HTS frequently disturbs cellular homeostasis and can result in drastic reductions in the growth, development of plants, which can lead to death [11, 12]. In this study, to examine the effects of HTS on the morphology and physiology of maca, two-week-old seedlings were treated at 42 °C for 0, 3, 6, 12 or 24 h. Seedlings grown at 25 °C for the designated time points were used as controls. As shown in Fig. 1a, the leaves of the seedlings exhibited slight chlorosis and badly wilted with prolonged HTS treatment for 12 and 24 h. This finding is consistent with previous phenotypes observed in other plant species subjected to HTS [17, 24, 25], indicating that HSRs were successfully induced. To validate the morphological phenotypes displayed in Fig. 1a, we also measured chlorophyll, malondialdehyde and soluble sucrose contents, as well as total antioxidant capacity in maca seedlings under HTS. A significant decrease in chlorophyll content in the maca seedling leaves was detected under HTS after 24 h, whilst there was no significant difference in chlorophyll content in the maca seedlings exposed to HTS for 0–12 h (Fig. 1b). Malondialdehyde is a product of membrane lipid peroxidation and increasing malondialdehyde content in cells indicates damage of plant cell membranes [26]. Compared with that in the control seedlings, malondialdehyde content was markedly increased in the maca seedlings following HTS for 12 and 24 h, and almost doubled for 24 h (Fig. 1c). As expected, the soluble sucrose content and total antioxidant capacity in the leaves of maca seedlings were dramatically increased as HTS treatment time progressed (Fig. 1d and e). These findings corroborate previous studies showing that plants grown under HTS have increased sucrose content and antioxidant capacity, which may be used by the plants to cope with HTS [27, 28].

### Proteomic expression profiles in maca seedlings under HTS

**Fig. 2:**
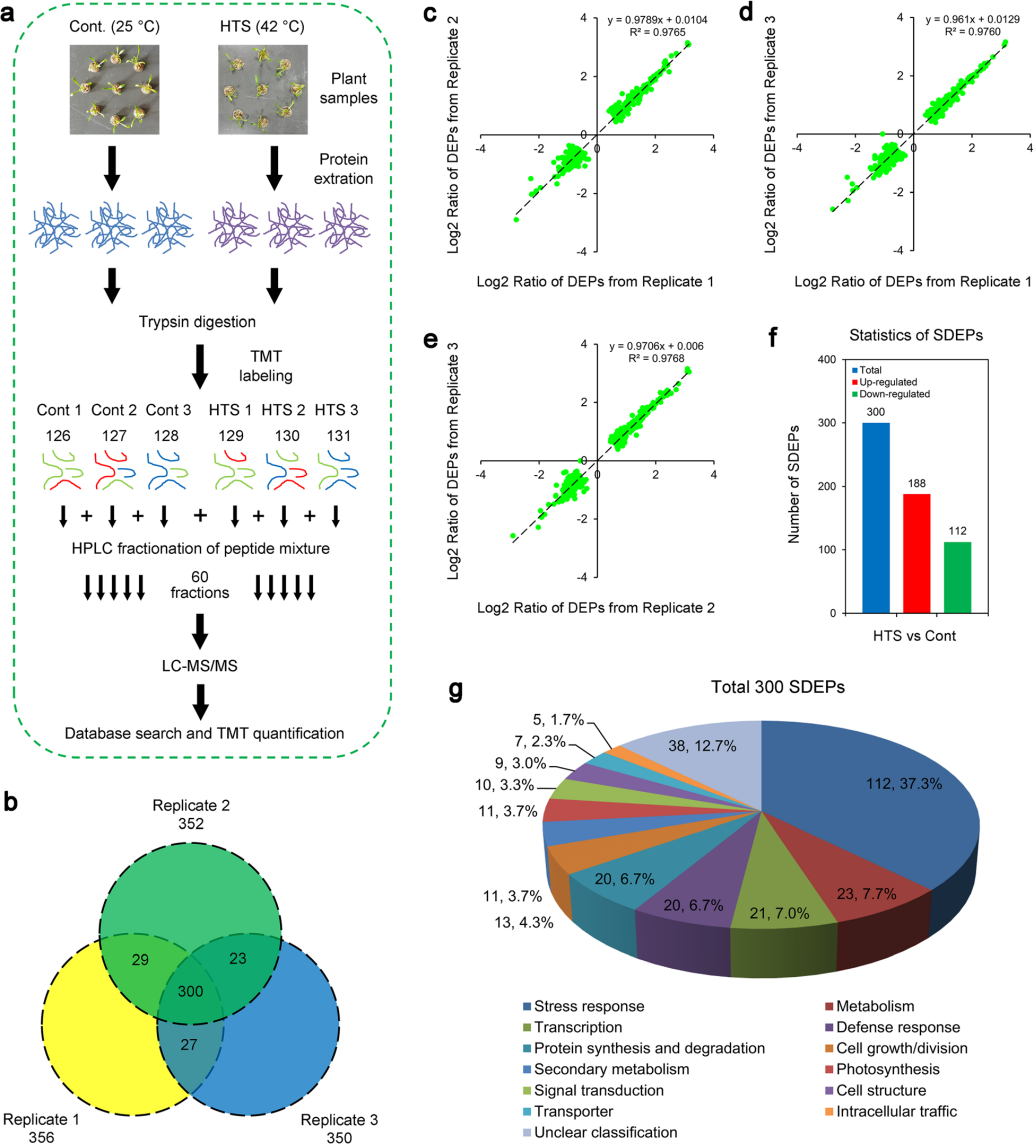
Proteomic analysis of two-week-old maca seedlings in response to HTS. **a** Experimental scheme for the proteomic analysis. Two-week-old maca seedlings treated with 42 °C or grown under control conditions (25 °C) for 12 h were used for the proteomic analysis. **b** Venn diagram analysis of differentially expressed proteins (DEPs) identified in maca seedlings following HTS. **c–e** Variance analyses of the DEPs from different biological replicates. **f** Numbers of the total, up-regulated and down-regulated DEPs in leaves of maca seedlings under HTS. **g** Functional classification of the significant DEPs (SDEPs)

To determine the early-stage proteomic alterations of maca in response to HTS, we employed a TMT-based proteomic approach (Fig. 2a) and explored the comprehensive protein profiles of maca seedlings grown under control conditions (25 °C) or HTS (42 °C) for 12 h. As a result, 55,426 individual peptides (Additional file 1) were obtained from 60,163 peptide spectra, which produced 6966 non-redundant protein species (Additional file 2) with a protein-level false discovery rate (FDR) at 1% [29, 30]. Compared with the control plants, 356, 352, and 350 proteins from biological replicates 1, 2, and 3, respectively, displayed differential alterations following HTS, sharing a subset of 300 proteins in all three replicates (Fig. 2b). Furthermore, a scatter plot analysis was performed to assess reproducibility of the three biological replicates. Notably, the regression slope from the linear regression analyses among different replicates reached 0.97 (Fig. 2c–e), suggesting that the TMT proteomic data are quite reproducible in the three biological replicates. Thus the DEPs detected in all three replicates were identified as significant DEPs (SDEPs) in our study. As a result, 300 SDEPs (Additional file 3) were identified in the leaves of maca seedlings following HTS, which included 188 up-regulated and 112 down-regulated proteins (Fig. 2f). Taken together, these results suggest that HTS causes a comprehensive change in proteome profiling of maca exposed to HTS for 12 h and, in turn, maca seedlings dramatically alter the levels of proteins putatively responding to HTS.

**Fig. 3:**
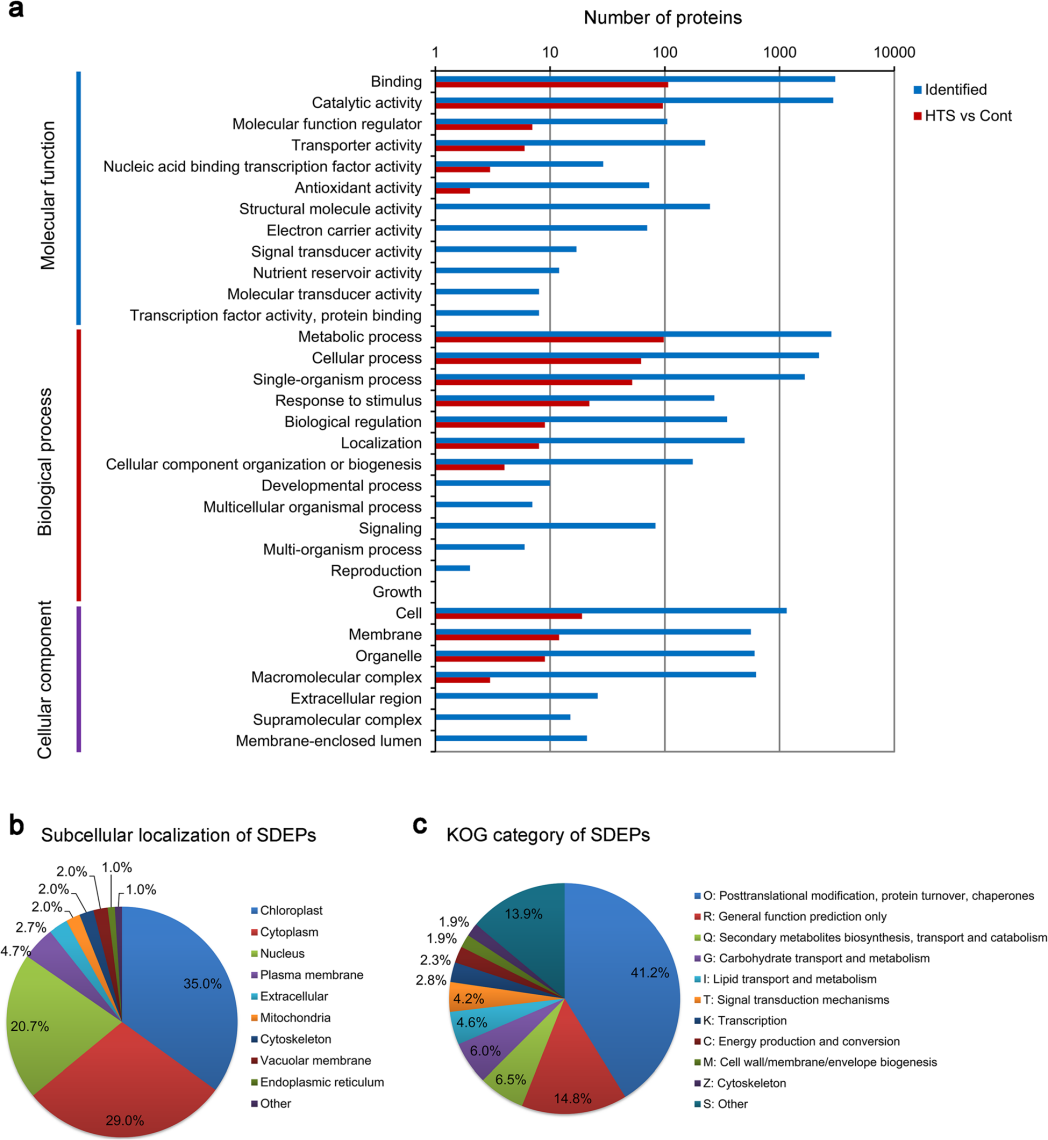
Classification information of the significant DEPs (SDEPs) of two-week-old maca seedlings following HTS (42 °C) for 12 h. Two-week-old maca seedlings grown at 25 °C for 12 h were used as controls. **a** Gene ontology (GO) analysis of all the identified proteins and SDEPs. **b** Subcellular localization analysis of the SDEPs. **c** Eukaryotic orthologous group (KOG) category classification of the SDEPs

Furthermore, a gene ontology (GO) analysis of the SDEPs was also performed as described previously [20]. As shown in Fig. 3a, the SDEPs were divided according to their GO terms into molecular function, cellular component and biological process. For molecular function, 35.7, 32.0, and 2.3% of the identified SDEPs were grouped under the terms ‘binding’, ‘catalytic activity’, and ‘molecular function regulator’, respectively (Fig. 3a). For biological process, 32.7, 20.7, and 17.3% of the identified SDEPs were the terms ‘metabolic process’, ‘cellular process’, and ‘single-organism process’, respectively (Fig. 3a). For cellular component, ‘cell’, ‘membrane’, and ‘organelle’ were the three most abundant terms, which accounted for 6.3, 4.0, and 3.0% of the SDEPs, respectively (Fig. 3a). The SDEPs were also grouped according to their predicted subcellular localizations. As shown in Fig. 3b, more than 9 subcellular components were identified and the SDEPs were mainly located in the chloroplast (35.0%), cytosol (29.0%), and nucleus (20.7%). A small number of SDEPs were located, for example, in the plasma membrane, extracellular, mitochondria, cytoskeleton, vacuolar membrane and endoplasmic reticulum (Fig. 3b). Moreover, using the eukaryotic orthologous group (KOG) classification, the SDEPs could be divided into 20 KOG functional categories. The three most highly represented categories were ‘posttranslational modification, protein turnover, chaperones’, ‘secondary metabolites biosynthesis, transport and catabolism’ and ‘carbohydrate transport and metabolism’, but, except for the category of ‘general function prediction only’. Interestingly, 89 SDEPs (29.7%) were classified into ‘posttranslational modification, protein turnover, chaperones’, which contained the most of the HTS-responsive proteins (Fig. 3c).

Since an aim of our proteomic analysis was to investigate the proteins in maca implicated in response to HTS and the associated response mechanisms, we classified the SDEPs based on the functional categories as described by Wang et al. [31]. As shown in Fig. 2g, the SDEPs had a broad range of important biological functions in stress response, metabolism, transcription, defense response, protein synthesis and degradation, cell growth/division, secondary metabolism, photosynthesis, signal transduction, cell structure, transporter, intracellular traffic and unknown functions. The SDEPs were found to be chiefly involved in stress response (37.3%), metabolism (7.7%), transcription (7.0%), defense response (6.7%), protein synthesis and degradation (6.7%), and cell growth/division (4.3%). These results align with previous proteomic studies which showed that HTS can up-regulate proteins involved in stress and defense, metabolism, protein synthesis and degradation, and cell growth/division in *Oryza sativa* [32], *Lycopersicon esculentum* [17] and *Pyropia haitanensis* [33]. Although the proteins identified in our study represent only a tiny proportion of the maca proteome, the identification of these HTS-responsive proteins may afford new insights into the response mechanisms of herbal plants to HTS. Some of the early-stage HTS-responsive proteins identified in the present study, which are implicated in the critical biological processes, are further discussed below.

#### Stress and defense responses

Plants have evolved various survival stress and defense responses to deal with environmental stresses [31, 34]. Plants frequently require a battery of genes/proteins participating in HSRs to resist the short-term high-temperature conditions [12, 35]. Previous studies have reported that heat shock proteins (HSPs) are major functional proteins induced by HTS [5, 35]. In the present study, we found 112 SDEPs implicated in the stress response of maca seedlings to HTS, 40 of which were HSP-related proteins (Additional file 3). Interestingly, in our TMT proteomic data, all of these 40 HSP-related proteins were found to be significantly up-regulated under HTS (Additional file 3). These findings are consistent with previous proteomic studies showing that a number of HSPs involved in HSRs are dramatically up-regulated following HTS [8, 33]. This indicates that maca seedlings initiate an extensive set of HSP-mediated HSRs during HTS and this may help plants to survive in high temperatures. It has been shown that the accumulation of ROS is another important HSR in plants during HTS [11, 12]. A dramatic increase in accumulation of ROS in apoplastic spaces can cause membrane lipid peroxidation and generate malondialdehyde [26]. This may be why the malondialdehyde content was increased in maca seedlings following HTS as described in Fig. 1c. Additionally, compared with the control seedlings, several ROS scavengers (Lmscaffold98.522, Lmscaffold290.57, Lmscaffold70.802, Lmscaffold141.198 and Lmscaffold309.514) were up-regulated more than 1.6-fold in maca seedlings following HTS (Additional file 3). The increased abundance of these proteins may account for the enhanced total antioxidant capacity in maca seedlings following HTS (Fig. 1e). This is in agreement with previous reports showing that the ROS scavengers are frequently induced by HTS at both transcript and protein levels [14, 36]. Furthermore, in the present study, a sucrose synthase (Lmscaffold452.187), which are implicated in in starch and sucrose metabolism [37], was up-regulated by approximately 1.8-fold in maca seedlings following HTS compared with the control plants (Additional file 3). The increased abundance of sucrose synthase may account for the elevated soluble sucrose content in maca seedlings following HTS described above (Fig. 1d). This observation aligns with previous studies reporting that sucrose synthase is frequently induced to maintain normal development and growth in plants under HTS [27, 38].

Beside the proteins associated with HSRs, 20 SDEPs related to defense responses were also identified in response to HTS, and nine of them were found to be dramatically increased following HTS. For example, the expression of a thionin (Lmscaffold352.114), two BCL-2-associated athanogenes (Lmscaffold251.178 and Lmscaffold358.358), a downy mildew resistance 6 (DMR6) (Lmscaffold467.737), and a Mal d 1-associated protein (Lmscaffold18.354) was > 2-fold higher in HTS-treated maca seedlings than in the control plants (Additional file 3). These suggest that HTS may have positive roles in the resistance of plants to biotic stresses such as pathogen attacks. For example, an adenosine kinase 1 (ADK1) (Lmscaffold237.395), which was up-regulated by approximately 1.9-fold according to our proteomic data, is an important component of innate antiviral defenses and frequently inactivated by geminivirus transcriptional activator proteins during viral infection [39, 40]. This finding is consistent with previous reports showing that elevated temperatures can enhance the antiviral defenses [41, 42].

#### Metabolism, secondary metabolism and photosynthesis

Previous reports have demonstrated that the down-regulation of metabolism is a generalized adaption response to HTS in plants [9, 33]. In the present study, 23 metabolism-related SDEPs were identified and 21 of them were decreased in maca seedlings following HTS (Additional file 3). This agrees with previous transcriptomic data showing that a specific cluster of genes, which are involved in the metabolism of carbohydrates, amino acids and lipids, are significantly decreased to adapt to the reduced need for primary metabolic products under HTS [15, 18]. Furthermore, 11 SDEPs involved in secondary metabolism were identified in the present study. Similar to the expression patterns of metabolism-related proteins, most of these secondary metabolism-associated proteins were down-regulated in maca seedlings in response to HTS (Additional file 3). These observations suggest that maca seedlings can alter their metabolic pathways via down-regulating the expression of proteins to allow them to survive HTS.

A decrease in photosynthesis is a known response to HTS [43]. Reduced levels of proteins implicated in the biosynthesis of chlorophylls and stabilization of photosynthetic systems have been reported in plants during HTS [8, 17, 32]. In this study, 11 SDEPs implicated in photosynthesis displayed significant alterations in abundance in maca seedlings following HTS. Based on their putative functions, seven of them (Lmscaffold696.41, Lmscaffold77.259, Lmscaffold106.673, Lmscaffold97.161, Lmscaffold804.148, Lmscaffold34.602, and Lmscaffold10.685) were involved in the biosynthesis of chlorophylls, whilst four of them (Lmscaffold344.261, Lmscaffold42.309, Lmscaffold695.324 and Lmscaffold455.183) were associated with the stabilization of photosynthetic systems. It is interesting to note that all of them were down-regulated under HTS (Additional file 3). The reduced abundance of these proteins may account for the reduced chlorophyll content in maca seedlings following HTS described above (Fig. 1b) and the perturbed photosynthetic machinery reported previously in other plants [9, 17, 33].

#### Transcription

Recent studies have demonstrated that a complex transcriptional regulatory network mediated by various transcriptional regulators is implicated in plant responses to HTS [5]. Among these, transcription factors have well established roles in HTS signaling and participate in regulating the expression of genes involved in HSRs [44]. In our study, 21 SDEPs associated with transcription were identified, and 15 of them were up-regulated in maca seedlings following HTS (Additional file 3). Among these up-regulated proteins, six of which were transcription factors that included a multiprotein bridging factor 1C (MBF1C) (Lmscaffold306.354), activation function 1 domain-containing protein (AF1) (Lmscaffold603.40), heat shock transcription factor A2 (HSFA2) (Lmscaffold26.42), WRKY transcription factor 70 (WRKY70) (Lmscaffold455.415), transcription factor DIVARICATA (Lmscaffold353.74) and transcription factor HY5 (HY5) (Lmscaffold9.304) (Additional file 3). In *Arabidopsis*, MBF1C was demonstrated to accumulate rapidly in leaves and act as a transcription factor to control the expression of 36 downstream genes during HTS [45]. In grape plants, both the mRNA and protein levels of MBF1C are reported to be significantly induced by HTS and play a crucial role in regulating the heat shock transcription factor-HSP pathway in the thermotolerance of grapes [8, 46]. In our TMT proteomic data, compared with the control seedlings, the MBF1C was up-regulated by approximately 3.9-fold in maca seedlings following HTS. This suggests that MBF1C is also a key regulator in controlling the HSRs of maca to HTS. Furthermore, a HSFA2 was also identified to be increased by approximately 2.6-fold in maca seedlings following HTS (Additional file 3). This finding aligns with previous studies reporting that HSFA2 is an important transcriptional regulator and is essential for HSRs in *Arabidopsis* [47] and tomato [48]. More interestingly, we also detected that the abundance of a HY5 increased by approximately 1.8-fold upon HTS (Additional file 3). To the best of our knowledge, this is the first time that HY5 has been identified to be implicated in HTS responses using a proteomic approach. HY5 is frequently believed to be involved in plant growth and development, protein degradation, and photomorphogenesis induced by both visible light and UV-B radiation, as well as in the biosynthesis of flavonoids induced by biotic and abiotic stresses [49]. We surmised that the up-regulation of maca HY5 may function to activate the flavonoid biosynthesis pathway to respond to HTS, because a recent report showed that it can reduce HTS-induced ROS accumulation and inhibition of pollen tube growth in tomato [50]. However, Delker et al. [51] reported that HY5 negatively controls the thermomorphogenesis of *Arabidopsis* to elevated temperatures via degradation of the basic-helix-loop-helix transcription factor phytochrome interacting factor 4, although there are cases where this effect is not evident [52, 53]. Thus, the exact role of HY5 in the HSRs of maca requires further examination in the future.

#### Protein synthesis and degradation

Previous studies have shown that protein synthesis and degradation are involved in regulating the HSRs of plants to HTS [9, 17, 36]. In the present study, we identified 20 SDEPs related to the protein synthesis or degradation (Additional file 3). Among these, 13 SDEPs (Lmscaffold34.282, Lmscaffold78.160, Lmscaffold36.276, Lmscaffold629.27, Lmscaffold45.1237, Lmscaffold195.283, Lmscaffold108.65, Lmscaffold1844.436, Lmscaffold152.33, Lmscaffold519.48, Lmscaffold234.199, Lmscaffold629.45 and Lmscaffold356.274 implicated in protein ubiquitination or proteolysis and two SDEPs (Lmscaffold37.599 and Lmscaffold127.69) involved in the regulation of protein translation increased their protein abundance in maca seedlings under HTS (Additional file 3). Interestingly, the accumulation levels of an ATP-dependent zinc metalloprotease FTSH 6 (FTSH6) (Lmscaffold629.27) and six caseinolytic protease B/HSP101 proteins (ClpB/HSP101s) (Lmscaffold34.282, Lmscaffold78.160, Lmscaffold195.283, Lmscaffold108.65, Lmscaffold1844.436, and Lmscaffold629.45) whose functions are related to proteolysis were increased more than 1.7-fold in maca seedlings following HTS, indicating that protein degradation is tightly controlled during HTS. Consistently, a recent study has demonstrated that *Arabidopsis* FTSH6 is induced by HTS and accumulates in the plastid where it joins with HSP21 to form a plastidial FTSH6-HSP21 control module to regulate thermomemory in plants [54]. Actually, several studies have reported that ClpB/HSP101s, which have an ATP-dependent Clp protease activity, are induced by HTS and have been implicated in the acquisition of thermotolerance in plants [55, 56]. Furthermore, we also found an ATP-dependent Clp protease ATP-binding subunit CLPT2 (Lmscaffold215.106), eukaryotic translation initiation factor 2A (Lmscaffold34.174), eukaryotic aspartyl protease family protein (Lmscaffold123.13), peptidase M1 family protein (Lmscaffold971.184), and a prolyl oligopeptidase family protein (Lmscaffold498.394) involved in protein translation and proteolysis were down-regulated in maca seedlings in response to HTS (Additional file 3). Taken together, these data suggest that the protein synthesis and degradation may have potential roles in the responses of maca seedlings to HTS. Moreover, in this study, a number of proteins associated with cell growth/division, signal transduction, cell structure, transporters and intracellular traffic were also identified (Additional file 3). Overall, our proteomic data provided here improve the understanding of the molecular mechanisms by which maca tolerates HTS, although the precise functions of these putative proteins still need to be further examined.

### Enrichment analysis of the SDEPs of maca seedlings in response to HTS

**Fig. 4:**
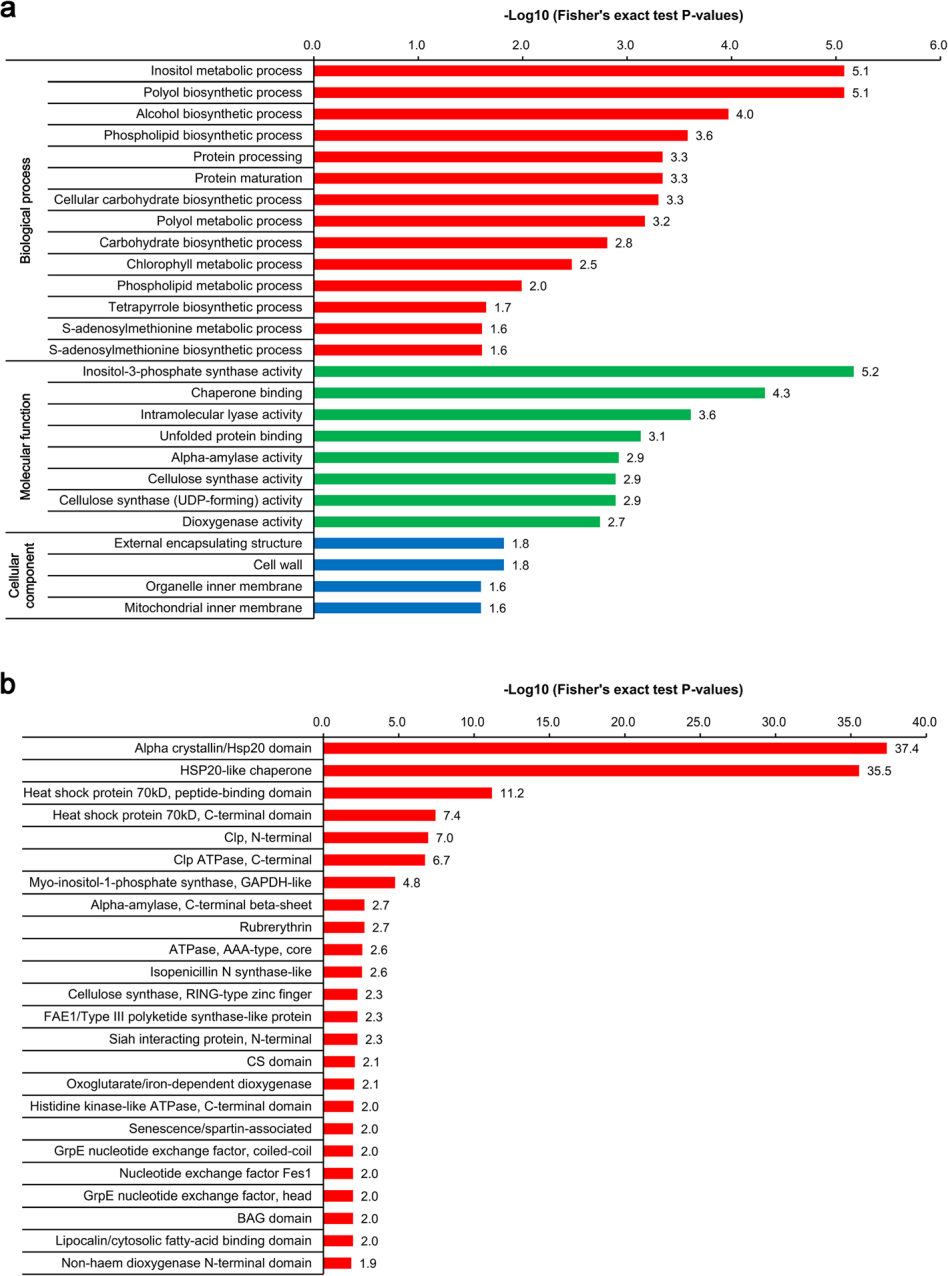
Gene ontology (GO) and protein domain enrichment analyses of the SDEPs in maca in response to HTS for 12 h. **a** GO enrichment analysis of the SDEPs. **b** Protein domain enrichment analysis of the SDEPs

To gain more information about the potential functions of these HTS-responsive SDEPs, a GO enrichment analysis was conducted as described previously [20]. As shown in Fig. 4a, 26 GO terms covering 227 SDEPs were enriched. For the biological process category, ‘inositol metabolic process’, ‘polyol biosynthetic process’ and ‘alcohol biosynthetic process’ were the three most significantly enriched GO terms; For the molecular function category, the top three enriched GO terms were ‘inositol-3-phosphate synthase activity’, ‘chaperone binding’ and ‘intramolecular lyase activity’. For the cellular component category, the most enriched GO terms were ‘external encapsulating structure’, ‘cell wall’ and ‘organelle inner membrane’. Furthermore, protein domain enrichment analysis showed that ‘alpha crystallin/hsp20 domain’, ‘hsp20-like chaperone’, ‘heat shock protein 70kD, peptide-binding domain’, ‘Heat shock protein 70kD, C-terminal domain’ and ‘clp, N-terminal’ were the top five significantly enriched domains (Fig. 4b).

Moreover, to further investigate the significantly changed metabolic pathways of maca plants under HTS, we also performed an enrichment analysis based on KEGG terms as described previously [31]. A Fisher’s exact test showed that 13 KEGG pathways were significantly enriched following HTS (Table 1). It is interesting that ‘protein processing in endoplasmic reticulum’, ‘porphyrin and chlorophyll metabolism’ and ‘linoleic acid metabolism’ were dramatically changed in maca seedlings following HTS (Table 1). Additionally, the pathways ‘sulfur metabolism’, ‘fatty acid elongation’, ‘cysteine and methionine metabolism’, ‘endocytosis’, ‘thiamine metabolism’, ‘betalain biosynthesis’, ‘inositol phosphate metabolism’, ‘steroid biosynthesis’, ‘ubiquinone and terpenoid-quinone biosynthesis’ and ‘RNA degradation’, also displayed *P*-values < 0.05 (Table 1).

**Table 1:** Representative HTS-responsive metabolic pathways enriched by KEGG pathway analysis in two-week-old maca seedlings exposed to HTS (42 °C) for 12 h

Serial no.	KEGG pathway^a^	KEGG ID	Mapping	Background	All mapping	All background	Fold enrichment	Fisher’s exact test *P*-values^b^
1	Protein processing in endoplasmic reticulum	ath04141	45	185	120	2859	5.80	7.85 × 10^−13^
2	Porphyrin and chlorophyll metabolism	ath00860	7	46	45	2859	9.67	1.55 × 10^−5^
3	Linoleic acid metabolism	ath00591	2	7	37	2859	22.08	3.29 × 10^−3^
4	Sulfur metabolism	ath00920	2	40	8	2859	17.87	5.07 × 10^−3^
5	Fatty acid elongation	ath00062	2	9	37	2859	17.177	5.54 × 10^−3^
6	Cysteine and methionine metabolism	ath00270	5	91	37	2859	4.25	5.70 × 10^−3^
7	Endocytosis	ath04144	4	83	26	2859	5.30	6.07 × 10^−3^
8	Thiamine metabolism	ath00730	2	17	26	2859	12.94	9.95 × 10^−3^
9	Betalain biosynthesis	ath00965	1	1	37	2859	77.27	1.29 × 10^−2^
10	Inositol phosphate metabolism	ath00562	3	33	49	2859	5.30	1.80 × 10^−2^
11	Steroid biosynthesis	ath00100	1	8	8	2859	44.67	2.22 × 10^−2^
12	Ubiquinone and terpenoid-quinone biosynthesis	ath00130	2	19	37	2859	8.13	2.43 × 10^−2^
13	RNA degradation	ath03018	3	84	26	2859	3.93	3.91 × 10^−2^

^a^All KEGG pathways were retrieved from KEGG release 88.2 on November 1, 2018

^b^Pathways were considered as significantly enriched at *P* < 0.05 and the pathways with a *P*-value higher than 0.05 were not listed

**Fig. 5:**
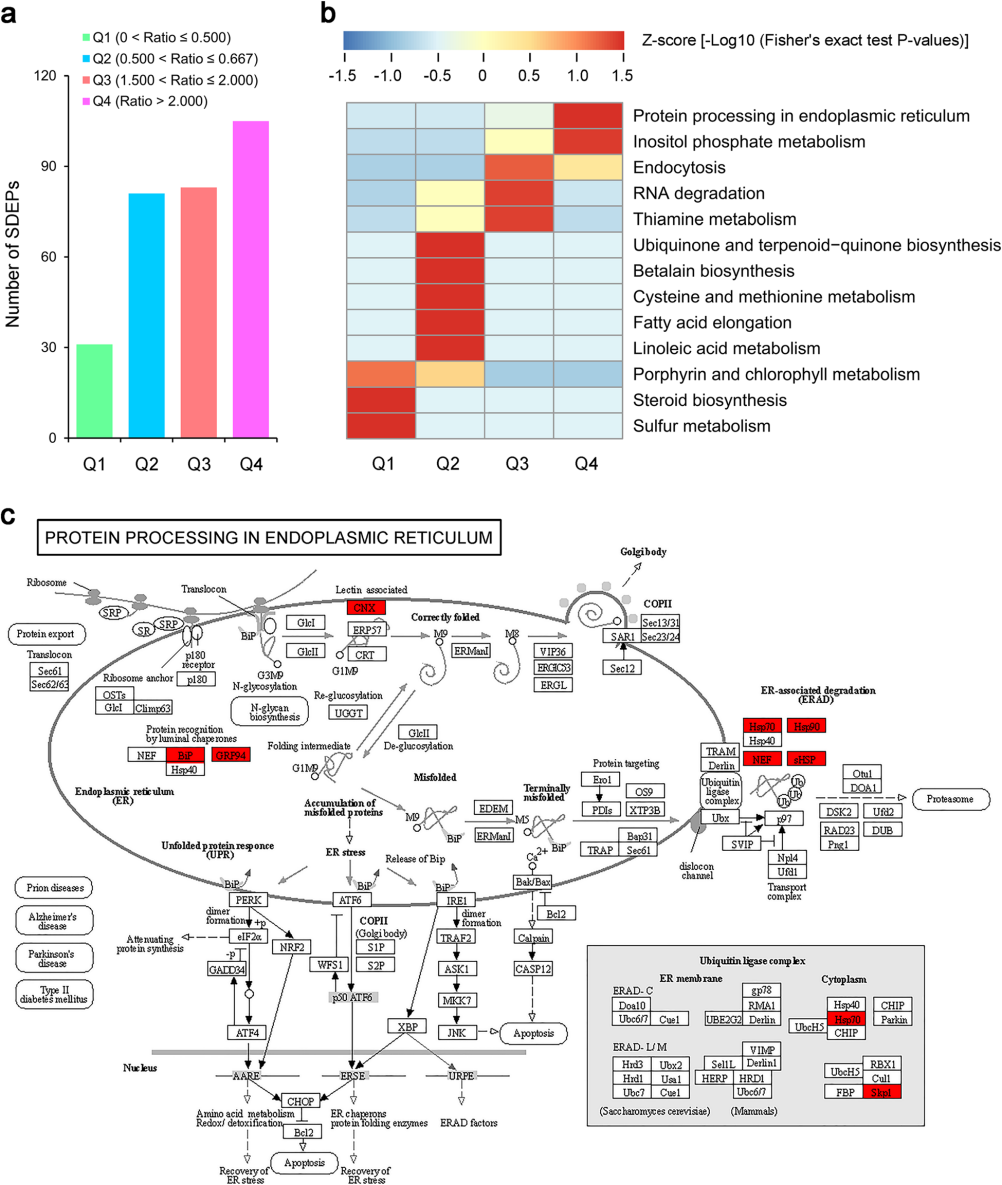
Kyoto Encyclopedia of Genes and Genomes (KEGG) enrichment analysis of the SDEPs responding to HTS for 12 h in maca. **a** Comparable groups of the SDEPs based on their quantification ratios. **b** Enriched KEGG pathways of the SDEPs in maca in response to HTS. **c** Schematic illustration of the SDEPs implicated in the ‘protein processing in endoplasmic reticulum’ pathway in maca. Red boxes indicate the SDEPs identified in maca under HTS

For cluster analysis, the SDEPs were classified into four groups according to their quantification ratios (Fig. 5a) and then subjected to KEGG-based enrichment. As shown in Fig. 5b, the SDEPs in Q1 (0 < ratio ≤ 0.500) were predominantly involved in ‘porphyrin and chlorophyll metabolism’, ‘sulfur metabolism’ and ‘steroid biosynthesis’; the SDEPs in Q2 (0.500 < ratio ≤ 0.667) were closely related to ‘porphyrin and chlorophyll metabolism’, ‘linoleic acid metabolism’, ‘fatty acid elongation’, ‘cysteine and methionine metabolism’, ‘betalain biosynthesis’ and ‘ubiquinone and terpenoid-quinone biosynthesis’; the SDEPs in Q3 (1.500 < ratio ≤ 2.000) were exclusively related to ‘protein processing in endoplasmic reticulum’, ‘endocytosis’, ‘thiamine metabolism’ and ‘RNA degradation’; and the SDEPs in Q4 (ratio > 2.000) were chiefly related to ‘protein processing in endoplasmic reticulum’ and ‘inositol phosphate metabolism’. These results indicate that the SDEPs divided into Q3 and Q4, which were up-regulated following HTS, are principally involved in ‘protein processing in endoplasmic reticulum’, ‘inositol phosphate metabolism’, ‘endocytosis’, ‘thiamine metabolism’ and ‘RNA degradation’, but especially in ‘protein processing in endoplasmic reticulum’. This is consistent with previous reports that several ‘protein processing in endoplasmic reticulum’ related genes/proteins are induced by HTS in various plant species [33, 36, 57]. A summary view of the ‘protein processing in endoplasmic reticulum’ pathway is shown in Fig. 5c.

### PPI networks for the SDEPs of maca seedlings in response to HTS

**Fig. 6:**
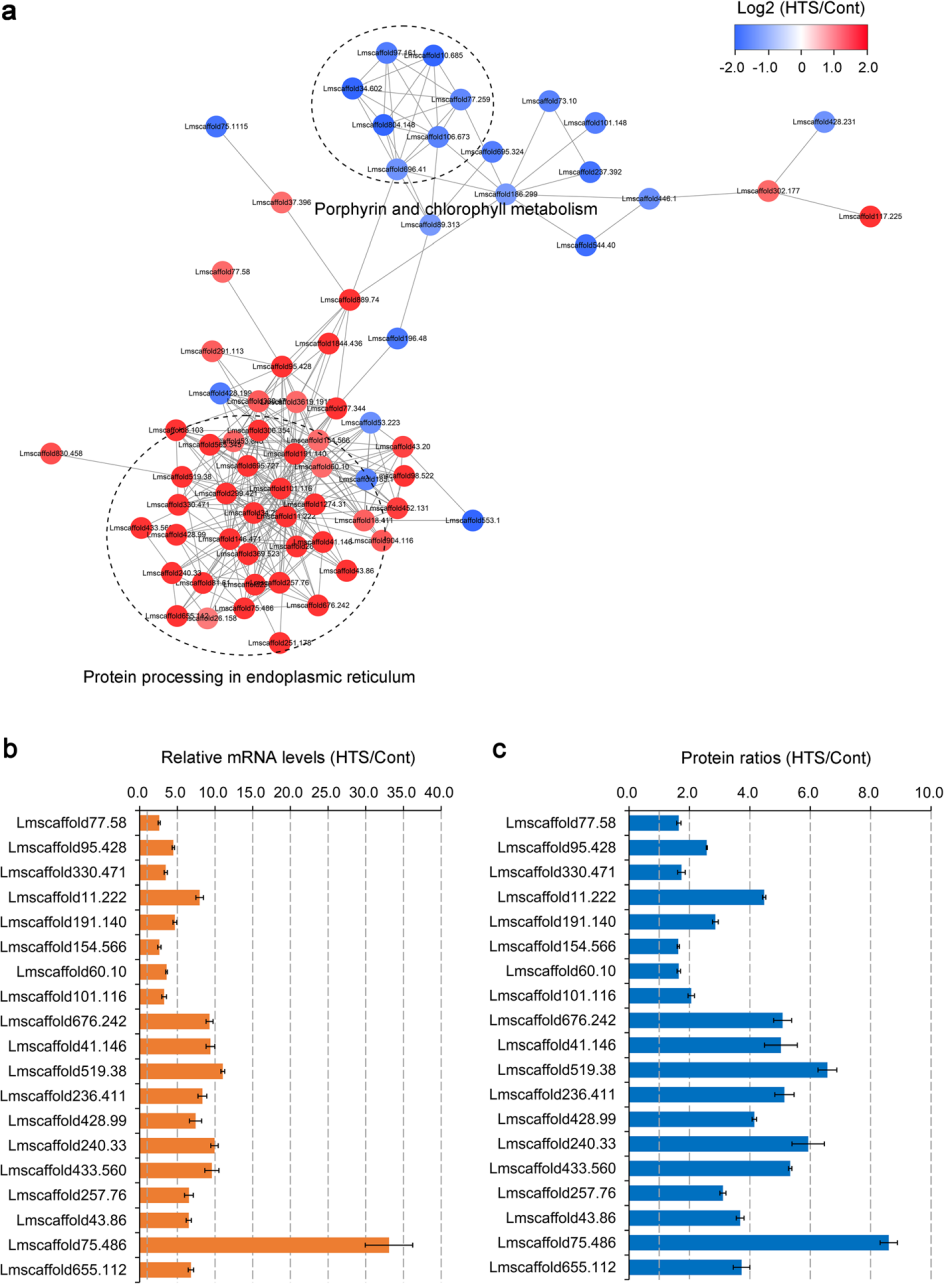
Protein-protein interaction (PPI) networks of the SDEPs and qRT-PCR analysis of gene expression levels. **a** PPI networks of the SDEPs were analyzed using the STRING database (v. 11.0) and visualized using the Cytoscape software (v. 3.6.1). The color bar represents gradients of Log2 transformed HTS/Cont ratios of the SDEPs. Red balls represent up-regulated SDEPs and blue balls represent down-regulated SDEPs. Black broken cycles represent the two enriched PPI clusters. **b** qRT-PCR analysis of gene expression levels in leaves of two-week-old maca seedlings exposed to HTS (42 °C) for 12 h. Two-week-old maca seedlings grown at 25 °C for 12 h were used as controls. Nineteen SDEPs implicated in the ‘protein processing in endoplasmic reticulum’ pathway were selected and subjected to qRT-PCR analysis using the same samples as for TMT proteomic data. *LmACT2* was used as an internal reference. Results are presented as means ± SD of three biological replicates and two technical replicates (*n* = 6). **c** Protein ratios of 19 SDEPs implicated in the ‘protein processing in endoplasmic reticulum’ pathway. Results are presented as means ± SD of the three proteomic data

PPI networks were further constructed to predict the potential biological functions of the HTS-responsive proteins in maca seedlings. A total of 69 SDEPs (47 up-regulated and 22 down-regulated, Additional file 4), which had confidence scores ≥ 0.7 (high confidence), were assigned to the PPI networks. As shown in Fig. 6a, two important cascades of biochemical processes, specifically ‘protein processing in endoplasmic reticulum’ and ‘porphyrin and chlorophyll metabolism’, were identified. Interestingly, most of the SDEPs implicated in the ‘protein processing in endoplasmic reticulum’ pathway had an increase in their accumulation following HTS (Fig. 6a and Additional file 5). In contrast, the SDEPs implicated in the ‘porphyrin and chlorophyll metabolism’ pathway were decreased (Fig. 6a and Additional file 6). These findings corroborate the above-mentioned results, which show that the ‘protein processing in endoplasmic reticulum’ pathway was significantly enhanced following HTS (Fig. 5b), and that there was an obvious decrease in chlorophyll content in the leaves of maca seedlings under HTS (Fig. 1b).

### qRT-PCR validation

Since the earlier TMT proteomic data, enrichment and PPI analyses showed that the ‘protein processing in endoplasmic reticulum’ pathway might play an essential role in HTS tolerance in maca seedlings (Figs. 5b and 6a, and Additional file 3), we further examined this pathway by determining the expression pattern of 19 related genes. Total RNA was extracted from leaves of maca seedlings grown under control conditions or HTS for 12 h, and subjected to qRT-PCR analysis. As shown in Fig. 6b, all of 19 SDEPs were significantly induced by HTS at the mRNA level and 17 of them were up-regulated more than 3-fold. These qRT-PCR results are in agreement with the TMT proteomic data (Fig. 6c and Additional file 3). This obvious up-regulation of proteins (genes) implicated in the ‘protein processing in endoplasmic reticulum’ pathway indicates a significant enhancement of protein biosynthesis, degradation and folding, which could be used by the plants to cope with HTS. These results suggest that the up-regulation of proteins correlates with their increase in the transcript abundance.

### A proposed pathway model of HTS responses in maca

**Fig. 7:**
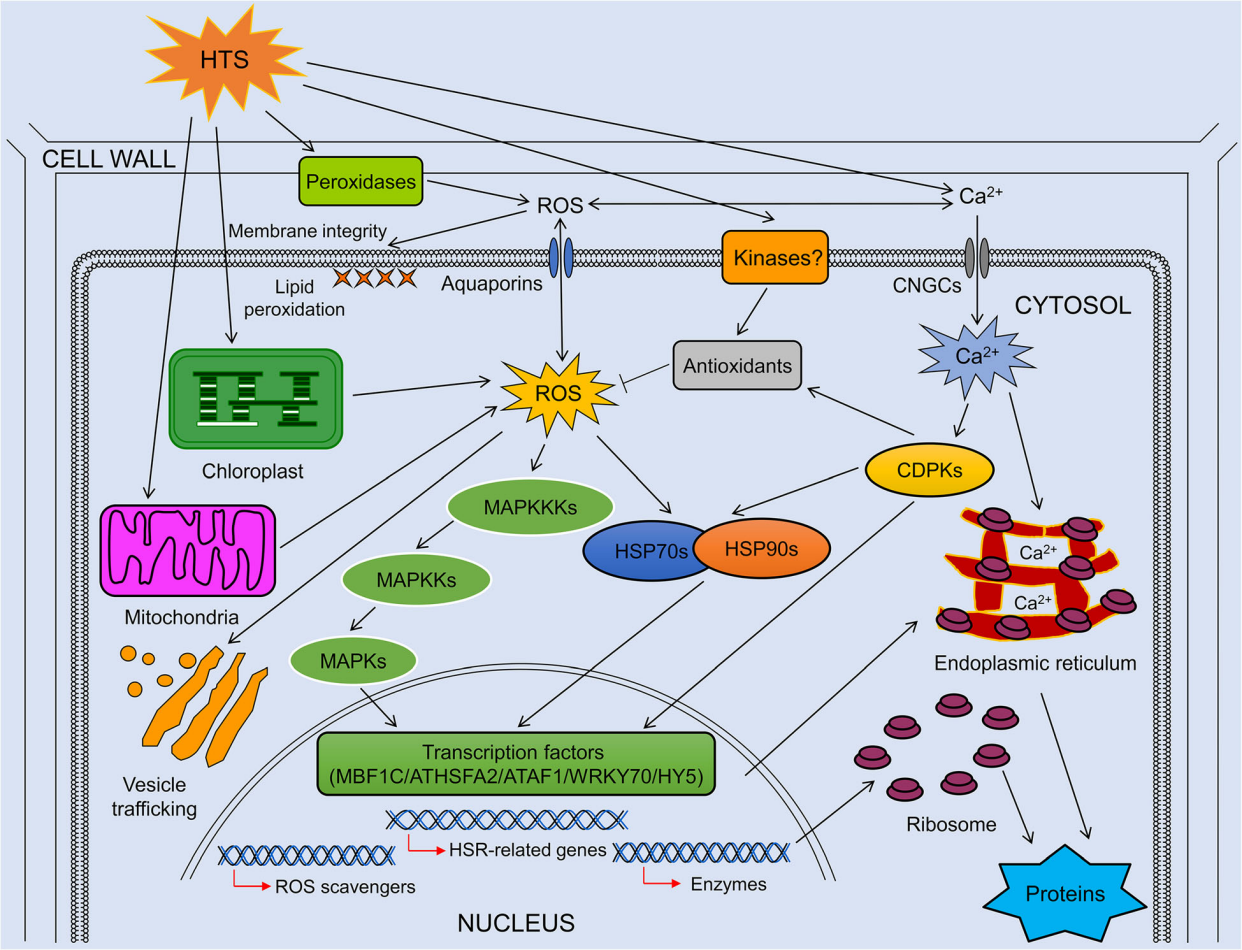
A proposed pathway model of HTS responses in maca. HTS might simultaneously destroy membrane integrity, induce malfunction of chloroplasts and mitochondria, and denature proteins in the cytosol or endoplasmic reticulum, leading to increased levels of cytosolic reactive oxygen species (ROS) and Ca^2+^, and activation of heat stress responses (HSRs). CNGC: cyclic-nucleotide gated channel; CDPK: Ca^2+^-dependent protein kinase; MAPK: mitogen-activated protein kinase

Using the results from our and previous studies [5, 12, 17, 36], we propose a putative synergistic regulatory network for maca that responds to HTS. As shown in Fig. 7, HTS can quickly stimulate peroxidase, chloroplast and mitochondria to generate ROS and cause an intracellular oxidative burst, which results in a series of physiological, metabolic and molecular alterations. First, high concentrations of ROS can cause membrane lipid peroxidation to produce malondialdehyde and destroy membrane integrity. Additionally, high concentrations of ROS can also lead to an increasing release of Ca^2+^ into the cytoplasm and activate Ca^2+^-dependent protein kinases (CDPKs). Second, to remove the excessively accumulated ROS, the antioxidant system is activated to maintain normal intracellular redox conditions via CDPKs or unknown kinases. Third, HSPs, which have key roles in regulating protein quality, are significantly up-regulated to protect proteins from being denatured by HTS. These HSPs are also able to regulate a variety of transcription factors such as MBF1C, HSFA2, AF1, WRKY70 and HY5, which control the expression of HSR-related genes, ROS scavengers, enzymes, and so on. Interestingly, a set of proteins implicated in protein processing in endoplasmic reticulum were also dramatically up-regulated in maca, and the result is consistent with the proteome responses of *Pyropia haitanensis* and *Ulva prolifera* to HTS [33, 36].

## Conclusions

Incubations at 42 °C for 12 or 24 h caused significant morphological and physiological changes in maca seedlings, and these were manifested as decreased chlorophyll content, increased malondialdehyde content, increased soluble sucrose content, and increased total antioxidant capacity. Proteomic analysis showed that the levels of 300 proteins were differentially changed by HTS for 12 h. Bioinformatics and qRT-PCR analyses indicated that protein processing in the endoplasmic reticulum is the most significantly enhanced KEGG pathway in maca in response to HTS for 12 h. These experimental data afford some new insights which may provide a deeper understanding of the molecular responses of herbal plants to HTS. These results also reveal that the combination of proteomics, bioinformatics and qRT-PCR analysis is a valid method to investigate response mechanisms to HTS in higher plants.

## Methods

### Plant material, culture and HTS treatment

Maca (*Lepidium meyenii* Walp) cultivar ‘Wumeng’ (breeding and preservation by Yunnan Agricultural University, YunR-SV-LM-045-2017) was utilized in this study. For cultivation, the seeds were sterilized with 1% (v/v) NaClO for 10 min, followed by five rinses in sterile water, and immersed in distilled water at 25 °C overnight. After germination, the seeds were immediately transferred to 60-well plates in 8-L plastic containers holding one-fifth-strength Hoagland solution (pH 5.5) [58], which was replenished every other day. The seedlings were grown in a green house at 25 °C under a 16/8 h (light/dark) photoperiod with a light intensity of 150–180 μE m^− 2^ s^− 1^. After pre-cultivation for 14 d, the maca plants were then transferred to 9-well plates in 1.2-L plastic pots containing fresh one-fifth-strength Hoagland solution (pH 5.5) for treatments. For the HTS treatment, the seedlings were grown in a growth chamber at 42 °C for 0, 3, 6, 12 or 24 h under continuous illumination at a light intensity of 150–180 μE m^− 2^ s^− 1^ as described previously [24, 59, 60]. Seedlings grown at 25 °C under same illumination for designated time points were used as controls. For the determination of chlorophyll and soluble sucrose contents, malondialdehyde concentration, and total antioxidant capacity, three biological replicates and three technical replicates were used. Each biological replicate was comprised of leaves from six two-week-old maca seedlings. For proteomic analysis, to reduce plant-to-plant variation, leaves from approximately 30–40 two-week-old maca seedlings were pooled into a mixed sample. Three independent sets of plants were collected to generate three biological replicates.

### Determination of chlorophyll and soluble sucrose contents

Chlorophyll was extracted from approximately 0.2 g of fresh leaves from six two-week-old maca seedlings exposed to HTS (42 °C) or grown under control conditions (25 °C) by grinding them in 2.5 mL of ice-cold 80% acetone and determined as described by Zhou et al. [61]. The chlorophyll content was spectrophotometrically measured as described by Lichtenthaler [62] and recorded as chlorophyll content per gram fresh weight (FW).

Soluble sucrose content was measured as described previously [63, 64]. In brief, approximately 0.2 g fresh leaves from six two-week-old maca seedlings exposed to HTS (42 °C) or grown under control conditions (25 °C) were powdered in liquid N_2_, homogenized in 2 mL of 80% (v/v) ethanol, heated in a water bath at 80 °C for 40 min, and centrifuged at 5000×g for 10 min at room temperature. For colorimetric determination, 0.25 mL of the 80% ethanol extract was added to 0.5 mL of water and digested with 0.5 mL 10% (w/v) aqueous KOH in a water bath at 100 °C for 3 min. The cooled reaction mixture was placed immediately in an ice-water bath, and then 2 mL of anthrone reagent (0.5 g of anthrone dissolved in 250 mL of 98% H_2_SO_4_) was added to the cooled mixture. The mixture was again incubated in a 100 °C water bath for 1 min, and then placed for 90 s in an ice-water bath. The sucrose concentration was determined at A_625_ using a UV-2450 spectrophotometer (Shimadzu, Japan) and recorded as soluble sucrose per gram FW. For the determination of chlorophyll and soluble sucrose contents, three biological replicates and three technical replicates were performed.

### Malondialdehyde concentration and total antioxidant capacity measurement

Malondialdehyde concentration was measured using the following formula: malondialdehyde (μmol g^− 1^ FW) = [6.45 × (A_532_ – A_600_) – 0.56 × A_450_] × V/W, in which A_532_, A_600_ and A_450_ are the absorbances at 532, 600 and 450 nm, respectively; and V is the volume of extraction; and W is the FW of the sample [65]. Total antioxidant capacity was determined using the ferric reducing antioxidant power (FRAP) assay as described previously [66]. The total antioxidant capacity was reported as μmol Fe^2+^ per gram FW. For the measurement of malondialdehyde concentration and total antioxidant capacity, three biological replicates and three technical replicates were performed.

### Protein extraction, trypsin digestion and TMT labeling

In this study, three independent sets of plants were collected to generate three biological replicates for each treatment. To reduce plant-to-plant variation, leaves from approximately 30–40 two-week-old maca seedlings were pooled into a mixed sample. Proteins from the maca leaf samples (approximately 1.0 g) were extracted with a lysis buffer (8 M urea, 1% Triton-100, 10 mM dithiothreitol and 1% protease inhibitor cocktail, pH 8.0) as described previously [31, 67].

For trypsin digestion and TMT labeling, the protein samples were digested with trypsin buffer (3 μg trypsin in 50 μL 0.1 M triethylammonium bicarbonate buffer at pH 8.5; Promega, USA) at 37 °C for 16 h and the digested peptides were collected by centrifugation and reconstituted in 0.5 M triethylammonium bicarbonate buffer (pH 8.5; Sigma, USA). And then, the digested peptides were labeled using the TMT Isobaric Mass Tagging Kit (Thermo Fisher Scientific, USA). In detail, the peptides from the control samples were labeled with TMT reagents 126, 127, and 128, and those from the HTS-treated samples were labeled with TMT reagents 129, 130, and 131 and incubated at 25 °C for 2 h before quenching with 8% hydroxylamine buffer (pH 8.0).

### High-performance liquid chromatography (HPLC) and liquid chromatography-tandem mass spectrometry (LC − MS/MS)

HPLC fractionation was conducted an Agilent 300Extend-C18 column (4.6 × 250 mm, 5 μm; Agilent Technologies, USA) as described by Zhan et al. [20]. Briefly, the peptides were first separated with a gradient of 8 to 32% acetonitrile (pH 9.0) at a flow rate of 0.5 mL min^− 1^ over 60 min into 60 fractions. Then, the collected 60 fractions were pooled into 18 ponds and dried by vacuum centrifugation.

LC − MS/MS was carried out with an automated Easy-nLC 1000 UPLC system coupled to a Q-Exactive™ Plus mass spectrometer (Thermo Fisher Scientific, USA) as described previously [30, 31]. In brief, the resulting peptides were dissolved in 0.1% formic acid (solvent A) and eluted using solvent B (0.1% formic acid in 98% acetonitrile) in a linear gradient comprised of an increase from 6 to 23% over 26 min, 23 to 35% in 8 min and climbing to 80% in 3 min then holding at 80% for the last 3 min on an Easy-nLC 1000 UPLC system. And then, the peptide samples were analyzed on a Q-Exactive™ Plus mass spectrometer (Thermo Fisher Scientific, USA) equipped with the NanoSpray Ionization (NSI) source coupled with a UPLC system. The working parameters of the mass spectrometer were selected according to previous studies [31, 68].

### Protein identification, quantification and reproducibility analysis

For protein identification, the resulting MS/MS spectra were searched against several protein databases containing reversed sequences from original databases, including the maca protein database (http://maca.eplant.org), NCBI protein database (https://www.ncbi.nlm.nih.gov/ protein/) and UniProt database (https://www.uniprot.org/) using MaxQuant software (v. 1.5.2.8) [69]. All reported data were reported based on 99% confidence for peptide and protein identification as described previously [29, 30].

The quantification ratio was calculated to assess the fold changes of the proteins identified in HTS-treated (HTS) vs control (Cont) plants. For a protein to be considered as significantly differentially expressed, it must have a fold-change ratio > 1.500 or < 0.667, be identified in all three biological replicates, and pass a student’s *t*-test adjusted by the Benjamini-Hochberg (BH) method [70] (*P*_*adj*_) < 0.05. Reproducibility analysis of the biological replicates was performed as described previously [31].

### Protein annotation, classification and enrichment analyses

GO and domain annotations were conducted as described by Zhan et al. [20]. In brief, GO annotation of the HTS-responsive proteome of maca was conducted by searching against the UniProt-GOA database (http://www.ebi.ac.uk/GOA). Those proteins that could not be annotated using the UniProt-GOA database were annotated by the InterProScan tool (http://www.ebi.ac.uk/interpro/) using, a protein sequence alignment method. Then, the GO annotations of the proteins were classified into three categories, including molecular function, biological process, and cellular component. Domain functional descriptions of the identified proteins were annotated by searching against the InterPro domain database using the InterProScan tool mentioned above. The Kyoto Encyclopedia of Genes and Genomes (KEGG) pathways of identified proteins were annotated by KEGG database using the KAAS tool (https://www.genome.jp/kaas-bin/kaas_main) and mapped using KEGG mapper (https://www.kegg.jp/kegg/mapper.html). The subcellular localizations of identified proteins were predicted using WoLF PSORT tool (https://www.genscript.com/psort/wolf_psort.html). The functional classification of SDEPs were carried out as described previously [31, 63].

For enrichment analysis, the SDEPs were classified into four subgroups according to their quantification ratios, and then each subgroup were subjected to KEGG-based enrichment as described by Wang et al. [31].

### Protein-protein interaction (PPI) analysis

All the SDEPs were searched against the STRING database (v. 11.0, http://string-db.org/ cgi/about.pl?footer_active_subpage = references) for PPI predictions. Interactions between proteins belonging to the data set were selected. Only the interactions having confidence scores ≥ 0.7 (high confidence) were fetched. The interaction network from STRING was visualized in Cytoscape (v. 3.6.1) [71].

### qRT-PCR analysis

For qRT-PCR analysis, three biological replicates were used and each replicate was comprised of leaves from six two-week-old maca seedlings exposed to HTS (42 °C) or grown under control conditions (25 °C) for 12 h. RNA extraction was carried out as described by Wang et al. [72]. Briefly, total RNA from leaf samples was isolated using an OminiPlant RNA Kit (CWBio, China) and genomic DNA contamination was cleaned by DNase I. cDNA was reverse transcribed from 1 μg of total RNA using ReverTra Ace qPCR Master Mix (Toyobo, Japan) following the manufacturer’s instructions. qRT-PCR was performed on an CFX96 Touch Deep Well Real-Time PCR Detection System (Bio-Rad, USA) as described previously [30, 63]. For each candidate gene, the PCR reactions were performed twice for each biological replicate and relative mRNA expression levels were calculated using the comparative *C*_T_ method [73]. *Maca actin 2* (*LmACT2*) was used as an internal control [74]. The primers used for qRT-PCR analysis are listed in Additional file 7. Raw data used for qRT-PCR analysis are provided in Additional file 8. The results were averages of three biological replicates.

### Statistical analysis

The data represent means ± standard deviation (SD) of three independent biological replicates. Student’s *t*-test was performed in Microsoft Excel (v.2016, Microsoft Corp., USA) and a BH-adjusted [70] *P*-value less than 0.05 (*P*_*adj*_ < 0.05) was considered statistically significant as described previously [31].

## Supplementary information

**Additional file 1 Table S1.** List of 55,426 peptides identified in this study.

**Additional file 2 Table S2.** List of 6966 proteins identified using TMT-based proteomic approach in this study.

**Additional file 3 Table S3.** List of identified proteins significant differentially expressed in two-week-old maca seedlings under high-temperature stress (HTS) for 12 h.

**Additional file 4 Table S4.** List of 69 differentially expressed proteins that were used for protein-protein interaction analysis.

**Additional file 5 Table S5.** List of 45 differentially expressed proteins that were involved in the protein processing in endoplasmic reticulum pathway.

**Additional file 6 Table S6.** List of 7 differentially expressed proteins that were involved in the porphyrin and chlorophyll metabolism pathway.

**Additional file 7 Table S7.** List of primers used in this study.

**Additional file 8 Table S8.** Raw data used for qRT-PCR analysis in this study.

## Data Availability

The datasets supporting the conclusions of this article are included within the article and its additional files. The mass spectrometry proteomics data reported in this paper have been deposited in the PRoteomics IDEntifications (PRIDE) database (http://www.ebi.ac.uk/pride/) (accession number: PXD021042).

## Abbreviations

AF1: Activation function 1 domain-containing protein
BH: Benjamini-Hochberg method
CDPK: Ca^2+^-dependent protein kinases
ClpB/HSP101: Caseinolytic protease B/HSP101 protein
CNGC: Cyclic-nucleotide gated channel
DEP: Differentially expressed protein
FDR: False discovery rate
FRAP: Ferric reducing antioxidant power
FW: Fresh weight
GO: Gene Ontology
HPLC: High-performance liquid chromatography
HSFA2: Heat shock transcription factor A2
HSP: Heat shock proteins
HSR: Heat stress response
HTS: High-temperature stress
HY5: Transcription factor HY5
KEGG: Kyoto Encyclopedia of Genes and Genomes
KOG: Eukaryotic orthologous group
LC − MS/MS: Liquid chromatography-tandem mass spectrometry
MAPK: Mitogen-activated protein kinase
MBF1C: Multiprotein bridging factor 1C
PPI: Protein-protein interaction
qRT-PCR: Quantitative RT-PCR
ROS: Reactive oxygen species
SD: Standard deviation
SDEP: Significant differentially expressed protein
TMT: Tandem mass tag
XTH: Xyloglucan endotransglucosylase/hydrolase
WRKY70: WRKY transcription factor 70.
